# Bis(μ-4-amino-3,5-dimethyl-4*H*-1,2,4-triazole)bis­[diiodidozinc(II)]

**DOI:** 10.1107/S160053681004852X

**Published:** 2010-12-04

**Authors:** Rongxian Zhang, Qiuyun Chen, Xiaofei Yang, Xiangyang Wu

**Affiliations:** a College of Chemistry and Chemical Engineering, Jiangsu University, Zhenjiang 212013, People’s Republic of China; b College of Material Science and Engineering, Jiangsu University, Zhenjiang 212013, People’s Republic of China; c School of Chemistry and Chemical Engineering, Jiangsu University, Zhenjiang 212013, People’s Republic of China

## Abstract

In the title compound, [Zn_2_I_4_(C_4_H_8_N_4_)_2_], the Zn^II^ atom is coordinated in a distorted tetra­hedral geometry by two N atoms from the triazole rings of two 4-amino-3,5-dimethyl-4*H*-1,2,4-triazole (admt) ligands and two iodide ligands. Doubly bridging admt ligands connect two Zn^II^ atoms, forming a centrosymmetric dimer. Weak N—H⋯I and C—H⋯I hydrogen bonds play an important role in the inter­molecular packing.

## Related literature

For background to transition metal complexes of 1,2,4-triazole derivatives, see: Liu *et al.* (1999 ▶, 2003 ▶); Zhao *et al.* (2002 ▶); Yi *et al.* (2004 ▶); Lavrenova *et al.* (1992 ▶); Haasnoot (2000 ▶); Zhang *et al.* (2007 ▶).
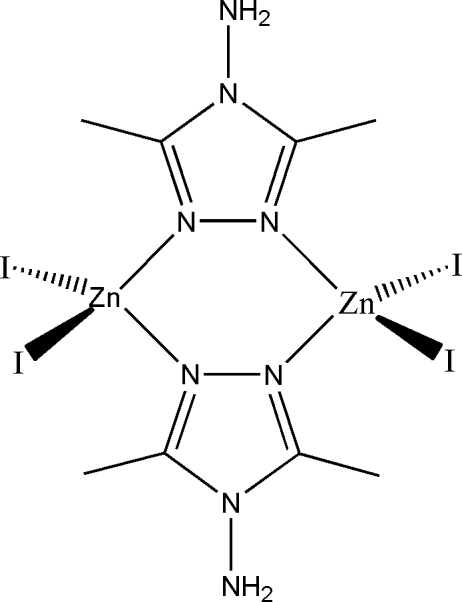


## Experimental

### 

#### Crystal data

[Zn_2_I_4_(C_4_H_8_N_4_)_2_]
*M* *_r_* = 862.63
Monoclinic, 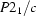

*a* = 7.4674 (19) Å
*b* = 13.442 (3) Å
*c* = 11.412 (3) Å
β = 102.598 (6)°
*V* = 1117.9 (5) Å^3^
*Z* = 2
Mo *K*α radiation
μ = 7.68 mm^−1^
*T* = 293 K
0.30 × 0.20 × 0.20 mm

#### Data collection

Rigaku Mercury CCD diffractometer
Absorption correction: multi-scan (*REQAB*; Jacobson, 1998 ▶) *T*_min_ = 0.207, *T*_max_ = 0.309
10214 measured reflections
2038 independent reflections
1760 reflections with *I* > 2σ(*I*)
*R*_int_ = 0.030

#### Refinement

*R*[*F* ^2^ > 2σ(*F* ^2^)] = 0.040
*wR*(*F* ^2^) = 0.098
*S* = 1.04
2038 reflections
108 parameters
2 restraints
H atoms treated by a mixture of independent and constrained refinement
Δρ_max_ = 1.35 e Å^−3^
Δρ_min_ = −1.11 e Å^−3^

### 

Data collection: *CrystalClear* (Rigaku, 2000 ▶); cell refinement: *CrystalClear*; data reduction: *CrystalClear*; program(s) used to solve structure: *SHELXS97* (Sheldrick, 2008 ▶); program(s) used to refine structure: *SHELXL97* (Sheldrick, 2008 ▶); molecular graphics: *SHELXTL* (Sheldrick, 2008 ▶); software used to prepare material for publication: *SHELXTL*.

## Supplementary Material

Crystal structure: contains datablocks global, I. DOI: 10.1107/S160053681004852X/zl2313sup1.cif

Structure factors: contains datablocks I. DOI: 10.1107/S160053681004852X/zl2313Isup2.hkl

Additional supplementary materials:  crystallographic information; 3D view; checkCIF report

## Figures and Tables

**Table 1:** Hydrogen-bond geometry (Å, °)

*D*—H⋯*A*	*D*—H	H⋯*A*	*D*⋯*A*	*D*—H⋯*A*
N4—H4*A*⋯I2^i^	0.86 (2)	2.98 (5)	3.706 (7)	144 (7)
N4—H4*A*⋯I1^ii^	0.86 (2)	3.23 (8)	3.720 (7)	119 (7)
N4—H4*B*⋯I1^iii^	0.86 (2)	3.27 (4)	4.090 (7)	161 (7)
C3—H3*A*⋯I1^iv^	0.96	3.24	3.930 (8)	130
C3—H3*B*⋯I1^iii^	0.96	3.43	3.888 (8)	112

Symmetry codes: (i) 
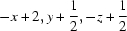
; (ii) 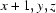; (iii) 
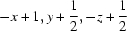
; (iv) 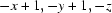.

**Fig. 1.**
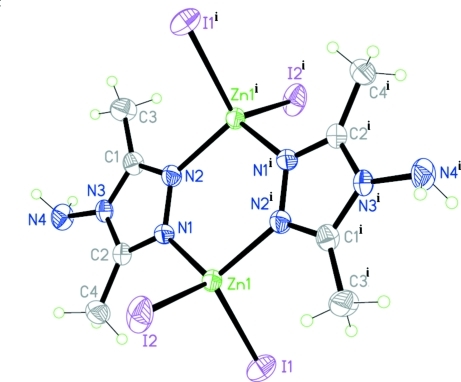
The dimeric structure of the title compound, showing the atom-numbering scheme. Displacement ellipsoids are drawn at the 30% probability level. [Symmetry codes: (i) -x + 1, -y + 1, -z.]

**Fig. 2.**
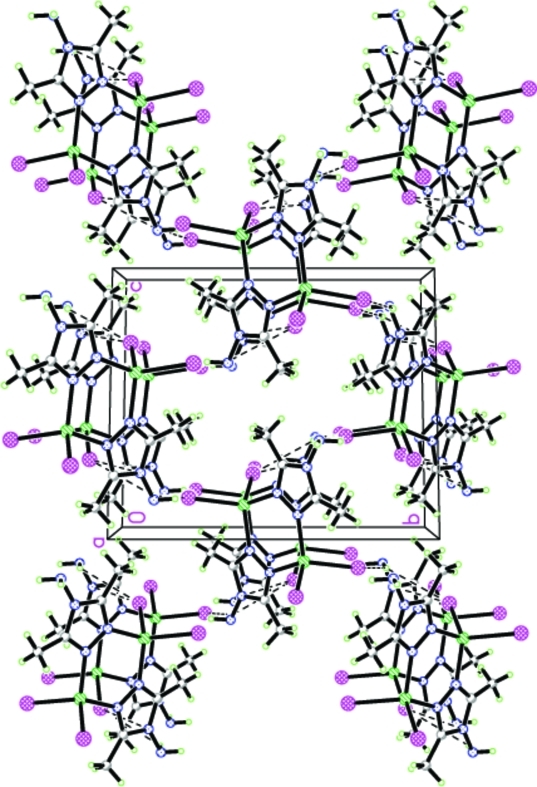
Cell packing plot of the title compound. The dashed lines represent N—H···I hydrogen bond interactions.

## supplementary crystallographic information

### Comment

1,2,4-Triazole and its derivatives are very interesting ligands because they combine the coordination geometry of both pyrazole and imidazole with regard to the arrangement of their three heteroatoms. A large number of mononuclear, oligonuclear and polynuclear transition metal complexes of 1,2,4-triazole derivatives have been synthesized and characterized due to their magnetic properties and novel topologies (Haasnoot, 2000). For 4-amino-3,5-dimethyl-1,2,4-triazole (admt), several Mn^II^ (Liu *et al.*, 1999), Co^II^, Ni^II^ (Zhao *et al.*, 2002), Cu^II^ (Liu *et al.*, 2003) and Cd^II^ compounds (Yi *et al.*, 2004) were synthesized. However, to best of our knowledge, only one Zn^II^-admt compound, [Zn_2_(admt)_2_Cl_4_], was synthesized (Lavrenova *et al.*, 1992). Here, we report the preparation and crystal structure of a dimeric Zn^II^ complex of [Zn(admt)I_2_]_2_.

The structure of the title compound is made up of neutral dimeric metallacycle. THe title compound has the same molecular structure as its chloro derivative [Zn_2_(admt)_2_Cl_4_], but the two compounds have different packing patterns and are not isotructural (Lavrenova *et al.*, 1992). In each dimeric metallacycle, as shown in Fig. 1, two zinc^II^ centers are connected by two admt ligands, resulting in a discrete Zn_2_(admt)_2_ 6-membered metallacycle which represents the smallest closed cyclic structure with a 1:1 metal-to-ligand ratio. Two triazole rings are coplanar. Each zinc^II^ center is four-coordinated with two nitrogen donors of two admt ligands (Zn1—N1 2.013 (5) Å; Zn1—N2^i^ (symmetry code i: -*x* + 1,-*y* + 1,-*z*) 2.046 (5) Å)and two I^-^ anions ligands (Zn1—I1 2.560 (1) Å; Zn1—I2 2.549 (1) Å), forming a distorted tetrahedral geometry. The Zn—N (triazole) bond lengths in the title compound are consistent with values in other Zn-triazole complexes (Zhang *et al.*, 2007; Lavrenova *et al.*, 1992). The N—Zn—N, N—Zn—I and I—Zn—I bond angles in the title compound are in the range of 106.8 (2) to 113.75 (3)°, near to the ideal tetrahedral value of ca. 109.5°.

The ligand admt is a 4-substituted 1,2,4-triazole and exhibits in the title compound the N1,N2-bidentate bridging coordination mode. Two admt ligands bridge two Zn(II) atoms to form a dimer with a Zn···Zn distance of 3.803 (2) Å. For a 4-substituted 1,2,4-triazole, by blocking the N4 donor position through substitution, only the N1 monodentate and N1,N2-bidentate coordination modes are possible. The N1,N2-bidentate coordination mode in the dimer has been observed.

There are weak hydrogen bonding interactions between the hydrogen atom of the amino NH_2_ group and the I^-^ anion of adjacent dimers (N4···I2^ii^ = 3.706 (7) Å; N4···I1^iii^ = 3.720 (7) Å; N4···I1^iv^ = 4.090 (7) Å) (symmetry codes: ii = -*x*+2, *y*+1/2, -*z*+1/2; iii = *x*+1, *y*, *z*; iv = -*x*+1, *y*+1/2, -*z*+1/2. There are also weak inter-dimer hydrogen bonding interactions between methyl hydrogen atoms and I^-^ anions (C3···I1^i^ = 3.930 (8) Å; C3···I1^iv^ = 3.888 (8) Å). These hydrogen bonding interactions do direct the packing of the crystal structure of the title compound (Fig. 2). No obvious π-π stacking interactions between the triazole rings is observed.

### Experimental

A 15 ml aqueous solution of 4-amino-3,5-dimethyl-1,2,4-triazole (admt) (1.0 mmol) was added to a 10 ml aqueous solution of Zn(NO_3_)_2_.6H_2_O (1.0 mmol) and KI (2.0 mmol) with stirring. The resultant solution was stored at room temperature and colourless crystal were obtained after about two weeks. Anal. Calcd. for C_8_H_16_I_4_N_8_Zn_2_: C, 11.14; H, 1.87; N, 12.99%. Found: C, 11.09; H, 1.83; N, 12.93%.

### Refinement

The H atoms of the amino group were obtained from difference Fourier maps and were refined with N—H distances of 0.86Å and *U*_iso_(H) = 1.2*U*_eq_(N). All other H atoms were placed in idealized positions and refined as riding with C—H distances of 0.96Å and *U*_iso_(H) = 1.5*U*_eq_(C).

### Crystal data

**Table:**

[Zn_2_I_4_(C_4_H_8_N_4_)_2_]	*F*(000) = 784
*M**_r_* = 862.63	*D*_x_ = 2.563 Mg m^−^^3^
Monoclinic, *P*2_1_/*c*	Mo *K*α radiation, λ = 0.71070 Å
Hall symbol: -P 2ybc	Cell parameters from 3727 reflections
*a* = 7.4674 (19) Å	θ = 3.0–25.4°
*b* = 13.442 (3) Å	µ = 7.68 mm^−^^1^
*c* = 11.412 (3) Å	*T* = 293 K
β = 102.598 (6)°	Block, colorless
*V* = 1117.9 (5) Å^3^	0.30 × 0.20 × 0.20 mm
*Z* = 2	

### Data collection

**Table:**

Rigaku Mercury CCD diffractometer	2038 independent reflections
Radiation source: fine-focus sealed tube	1760 reflections with *I* > 2σ(*I*)
graphite	*R*_int_ = 0.030
Detector resolution: 7.31 pixels mm^-1^	θ_max_ = 25.4°, θ_min_ = 3.0°
ω scans	*h* = −8→8
Absorption correction: multi-scan (Jacobson, 1998)	*k* = −16→14
*T*_min_ = 0.207, *T*_max_ = 0.309	*l* = −13→13
10214 measured reflections	

### Refinement

**Table:**

Refinement on *F*^2^	Primary atom site location: structure-invariant direct methods
Least-squares matrix: full	Secondary atom site location: difference Fourier map
*R*[*F*^2^ > 2σ(*F*^2^)] = 0.040	Hydrogen site location: inferred from neighbouring sites
*wR*(*F*^2^) = 0.098	H atoms treated by a mixture of independent and constrained refinement
*S* = 1.04	*w* = 1/[σ^2^(*F*_o_^2^) + (0.0483*P*)^2^ + 3.598*P*] where *P* = (*F*_o_^2^ + 2*F*_c_^2^)/3
2038 reflections	(Δ/σ)_max_ < 0.001
108 parameters	Δρ_max_ = 1.35 e Å^−^^3^
2 restraints	Δρ_min_ = −1.11 e Å^−^^3^

### Special details

**Table:**

Geometry. All e.s.d.'s (except the e.s.d. in the dihedral angle between two l.s. planes) are estimated using the full covariance matrix. The cell e.s.d.'s are taken into account individually in the estimation of e.s.d.'s in distances, angles and torsion angles; correlations between e.s.d.'s in cell parameters are only used when they are defined by crystal symmetry. An approximate (isotropic) treatment of cell e.s.d.'s is used for estimating e.s.d.'s involving l.s. planes.
Refinement. Refinement of *F*^2^ against ALL reflections. The weighted *R*-factor *wR* and goodness of fit *S* are based on *F*^2^, conventional *R*-factors *R* are based on *F*, with *F* set to zero for negative *F*^2^. The threshold expression of *F*^2^ > σ(*F*^2^) is used only for calculating *R*-factors(gt) *etc*. and is not relevant to the choice of reflections for refinement. *R*-factors based on *F*^2^ are statistically about twice as large as those based on *F*, and *R*- factors based on ALL data will be even larger.

### Fractional atomic coordinates and isotropic or equivalent isotropic displacement parameters (Å^2^)

**Table:**

	*x*	*y*	*z*	*U*_iso_*/*U*_eq_	
Zn1	0.42494 (9)	0.40526 (5)	0.10329 (6)	0.0386 (2)	
I1	0.16668 (7)	0.43239 (4)	0.21514 (5)	0.0657 (2)	
I2	0.56923 (7)	0.23271 (4)	0.13413 (6)	0.0743 (2)	
N1	0.6190 (7)	0.5117 (4)	0.1541 (4)	0.0405 (12)	
N2	0.6793 (7)	0.5755 (4)	0.0763 (5)	0.0417 (12)	
N3	0.8344 (7)	0.6026 (4)	0.2567 (4)	0.0387 (12)	
N4	0.9608 (9)	0.6397 (5)	0.3571 (6)	0.0565 (15)	
C1	0.8137 (9)	0.6303 (5)	0.1401 (6)	0.0439 (15)	
C2	0.7150 (8)	0.5286 (5)	0.2633 (5)	0.0391 (14)	
C3	0.9265 (11)	0.7049 (6)	0.0964 (7)	0.060 (2)	
H3A	0.9910	0.6741	0.0418	0.090*	
H3B	1.0133	0.7325	0.1631	0.090*	
H3C	0.8492	0.7569	0.0556	0.090*	
C4	0.7035 (11)	0.4766 (6)	0.3741 (6)	0.0603 (19)	
H4C	0.5954	0.4358	0.3600	0.090*	
H4D	0.6975	0.5244	0.4356	0.090*	
H4E	0.8101	0.4355	0.3993	0.090*	
H4A	1.069 (5)	0.632 (6)	0.345 (7)	0.072*	
H4B	0.963 (12)	0.7033 (17)	0.353 (8)	0.072*	

### Atomic displacement parameters (Å^2^)

**Table:**

	*U*^11^	*U*^22^	*U*^33^	*U*^12^	*U*^13^	*U*^23^
Zn1	0.0331 (4)	0.0396 (4)	0.0418 (4)	−0.0002 (3)	0.0052 (3)	0.0039 (3)
I1	0.0538 (3)	0.0680 (4)	0.0837 (4)	0.0007 (2)	0.0337 (3)	−0.0101 (3)
I2	0.0540 (3)	0.0465 (3)	0.1169 (5)	0.0152 (2)	0.0064 (3)	0.0084 (3)
N1	0.037 (3)	0.043 (3)	0.040 (3)	−0.004 (2)	0.006 (2)	0.007 (2)
N2	0.039 (3)	0.044 (3)	0.039 (3)	−0.004 (2)	0.003 (2)	0.001 (2)
N3	0.034 (3)	0.039 (3)	0.038 (3)	0.000 (2)	−0.001 (2)	−0.005 (2)
N4	0.051 (4)	0.063 (4)	0.048 (3)	−0.012 (3)	−0.005 (3)	−0.015 (3)
C1	0.046 (4)	0.039 (3)	0.042 (4)	−0.004 (3)	0.001 (3)	−0.006 (3)
C2	0.035 (3)	0.040 (3)	0.040 (3)	0.002 (3)	0.003 (3)	−0.002 (3)
C3	0.065 (5)	0.058 (4)	0.054 (4)	−0.028 (4)	0.008 (4)	−0.005 (3)
C4	0.060 (5)	0.072 (5)	0.046 (4)	−0.008 (4)	0.005 (3)	0.008 (4)

### Geometric parameters (Å, °)

**Table:**

Zn1—N1	2.029 (5)	N4—H4A	0.86 (2)
Zn1—N2^i^	2.044 (5)	N4—H4B	0.86 (2)
Zn1—I2	2.5493 (10)	C1—C3	1.465 (9)
Zn1—I1	2.5603 (10)	C2—C4	1.464 (9)
N1—C2	1.314 (8)	C3—H3A	0.9600
N1—N2	1.378 (7)	C3—H3B	0.9600
N2—C1	1.327 (8)	C3—H3C	0.9600
N2—Zn1^i^	2.044 (5)	C4—H4C	0.9600
N3—C2	1.349 (8)	C4—H4D	0.9600
N3—C1	1.358 (8)	C4—H4E	0.9600
N3—N4	1.408 (7)		
			
N1—Zn1—N2^i^	106.8 (2)	N2—C1—N3	107.2 (6)
N1—Zn1—I2	110.38 (15)	N2—C1—C3	128.0 (6)
N2^i^—Zn1—I2	108.08 (15)	N3—C1—C3	124.8 (6)
N1—Zn1—I1	109.00 (15)	N1—C2—N3	107.7 (5)
N2^i^—Zn1—I1	108.58 (16)	N1—C2—C4	127.9 (6)
I2—Zn1—I1	113.73 (3)	N3—C2—C4	124.4 (6)
C2—N1—N2	108.4 (5)	C1—C3—H3A	109.5
C2—N1—Zn1	126.9 (4)	C1—C3—H3B	109.5
N2—N1—Zn1	124.6 (4)	H3A—C3—H3B	109.5
C1—N2—N1	107.9 (5)	C1—C3—H3C	109.5
C1—N2—Zn1^i^	123.8 (4)	H3A—C3—H3C	109.5
N1—N2—Zn1^i^	128.2 (4)	H3B—C3—H3C	109.5
C2—N3—C1	108.8 (5)	C2—C4—H4C	109.5
C2—N3—N4	123.4 (5)	C2—C4—H4D	109.5
C1—N3—N4	127.8 (6)	H4C—C4—H4D	109.5
N3—N4—H4A	108 (6)	C2—C4—H4E	109.5
N3—N4—H4B	109 (6)	H4C—C4—H4E	109.5
H4A—N4—H4B	94 (8)	H4D—C4—H4E	109.5
			
N2^i^—Zn1—N1—C2	177.2 (5)	Zn1^i^—N2—C1—C3	−7.6 (10)
I2—Zn1—N1—C2	−65.6 (5)	C2—N3—C1—N2	1.6 (7)
I1—Zn1—N1—C2	60.0 (5)	N4—N3—C1—N2	179.7 (6)
N2^i^—Zn1—N1—N2	−6.8 (6)	C2—N3—C1—C3	−176.4 (7)
I2—Zn1—N1—N2	110.5 (4)	N4—N3—C1—C3	1.7 (11)
I1—Zn1—N1—N2	−124.0 (4)	N2—N1—C2—N3	0.5 (7)
C2—N1—N2—C1	0.5 (7)	Zn1—N1—C2—N3	177.1 (4)
Zn1—N1—N2—C1	−176.2 (4)	N2—N1—C2—C4	−177.8 (7)
C2—N1—N2—Zn1^i^	−175.0 (4)	Zn1—N1—C2—C4	−1.2 (10)
Zn1—N1—N2—Zn1^i^	8.3 (7)	C1—N3—C2—N1	−1.3 (7)
N1—N2—C1—N3	−1.3 (7)	N4—N3—C2—N1	−179.5 (6)
Zn1^i^—N2—C1—N3	174.5 (4)	C1—N3—C2—C4	177.1 (6)
N1—N2—C1—C3	176.7 (7)	N4—N3—C2—C4	−1.1 (10)

Symmetry codes: (i) −*x*+1, −*y*+1, −*z*.

### Hydrogen-bond geometry (Å, °)

**Table:**

*D*—H···*A*	*D*—H	H···*A*	*D*···*A*	*D*—H···*A*
N4—H4A···I2^ii^	0.86 (2)	2.98 (5)	3.706 (7)	144 (7)
N4—H4A···I1^iii^	0.86 (2)	3.23 (8)	3.720 (7)	119 (7)
N4—H4B···I1^iv^	0.86 (2)	3.27 (4)	4.090 (7)	161 (7)
C3—H3A···I1^i^	0.96	3.24	3.930 (8)	130.
C3—H3B···I1^iv^	0.96	3.43	3.888 (8)	112.

Symmetry codes: (ii) −*x*+2, *y*+1/2, −*z*+1/2; (iii) *x*+1, *y*, *z*; (iv) −*x*+1, *y*+1/2, −*z*+1/2; (i) −*x*+1, −*y*+1, −*z*.
